# Phytotoxic Metabolites Produce by *Fusarium oxysporum* f. sp. *cubense* Race 2

**DOI:** 10.3389/fmicb.2021.629395

**Published:** 2021-04-15

**Authors:** N. Portal González, A. Soler, C. Ribadeneira, J. Solano, Roxana Portieles, L. Herrera Isla, B. Companioni, Orlando Borras-Hidalgo, Ramon Santos Bermudez

**Affiliations:** ^1^School of Biological Science and Technology, University of Jinan, Jinan, China; ^2^Facultad de Ciencias Agropecuarias, Universidad Técnica Luis Vargas Torres de Esmeraldas, Esmeraldas, Ecuador; ^3^Centre de Coopération Internationale en Recherche Agronomique pour le Développement (Réunion), Saint-Pierre, Réunion; ^4^Universidad Estatal de Bolívar, Guaranda, Guaranda, Ecuador; ^5^Joint R&D Center of Biotechnology, RETDA, Yota Bio-Engineering Co., Ltd., Rizhao, China; ^6^Universidad Central Marta Abreu de Las Villas, Santa Clara, Cuba; ^7^Universidad Autónoma Agraria Antonio Narro, Saltillo, Mexico; ^8^State Key Laboratory of Biobased Material and Green Papermaking, Shandong Provincial Key Laboratory of Microbial Engineering, Qilu University of Technology, Jinan, China

**Keywords:** *Fusarium* wilt, panama disease, fusaric acid, phytotoxins, beauvericin, enniatin A

## Abstract

Banana is a major tropical fruit crop but banana production worldwide is seriously threatened due to *Fusarium* wilt. *Fusarium oxysporum* f. sp. *cubense* (*Foc*), the causal agent of *Fusarium* wilt of banana (also referred as Panama disease) is an asexual, soil inhabiting facultative parasite. *Foc* isolates can be classified into three races that are not defined genetically, but for their pathogenicity to different banana cultivars. Despite mycotoxins being some of the best studied virulence factors of phytopathogenic fungi and these have been useful for the prediction of *Foc* virulence on banana plants, toxins produced by *Foc* race 2 strains have not been previously identified. The aim of this contribution was to identify the phytotoxic metabolites closely related to banana wilt caused by a *Foc* race 2 strain. We used an *in vitro* bioassay on detached banana leaves to evaluate the specificity of the microbial culture filtrates before a partial purification and further identification of *Foc* race 2 phytotoxins. A 29-day-old host-specific culture filtrate was obtained but specificity of culture filtrate was unrecovered after partial purification. The non-specific phytotoxins were characterized as fusaric acid, beauvericin, and enniatin A. Whereas some, if not all, of these phytotoxins are important virulence factors, a proteinaceous fraction from the specific 29-day-old culture filtrate protected the leaves of the resistant banana cultivar from damage caused by such phytotoxic metabolites.

## Introduction

Banana is a major tropical fruit crop and the staple food of millions of people around the world. However, banana production worldwide is seriously threatened due to *Fusarium* wilt, previously referred as Panama disease, caused by *Fusarium oxysporum* f. sp. *cubense* [*F. oxysporum* Schlecht. f. sp. *cubense* (E.F. Smith) Snyd. and Hans.]. *Fusarium* wilt of banana is a classical vascular wilt disease (Ploetz, 2019). The vascular wilt fungus *Fusarium oxysporum* is an asexual, soil inhabiting facultative parasite (Li et al., 2019).

*Fusarium oxysporum* f. sp. *cubense* (*Foc*) can be classified into three races that are not defined genetically, but were determined by their degree of pathogenicity against different banana cultivars. Race 1 affects Gros Michel (AAA) and some AAB (Maqueño, Silk, Pome) or ABB (Pisang Awak) banana cultivars; race 2 affects the hybrid triploid cultivar “Bluggoe” (ABB), and other closely related cooking bananas, whereas race 4 causes disease to Cavendish banana cultivars (AAA) and to all banana cultivars susceptible to both race 1 and race 2. Race 4 isolates were further divided into subtropical race 4 (STR4) and tropical race 4 (TR4) based on their requirements of disease-predisposing conditions (Ploetz, 2015). *Foc* is genetically heterogeneous and possibly has had several origins (polyphyletic; Ploetz, 2019). According to vegetative compatibility, 24 vegetative compatibility groups (VCGs) have been identified in *Foc* isolates. Race 3 was previously reported to affect *Heliconia* spp. (formerly included in the family Musaceae) but is no longer considered as being pathogenic to bananas (Fourie et al., 2009).

A greater understanding of molecular mechanisms underlying the interaction among *Foc* isolates currently grouped in different races and host needs to be explored. As hemibiotrophic pathogen, *Foc* utilizes an array of virulence factors to help its infection of the host plants (Guo et al., 2014). Much effort has been made to understand pathogenesis in *Foc* (Widinugraheni et al., 2018; Li et al., 2019).

However, studies on the *Foc* infection process have been mainly based on susceptible banana varieties inoculated with *Foc* race 1, *Foc* race 4 (Liu et al., 2020), or both (Fei et al., 2019). A few fungal molecules that contribute to *Foc* pathogenicity have been described. These include the phytotoxin fusaric acid (FA), which has been isolated from both race 1 and 4 strains (Dong et al., 2012; Li et al., 2013; Liu et al., 2020), whereas beauvericin (BEA) was more detected from *Foc* strain representatives of race 1 (Li et al., 2013). In addition to these mycotoxins, enniatins (ENs) were substantially detected in *Foc* strains of 20 VCGs investigated by Shao et al. (2020) and Fumonisin B1 (FB1) was identified in a host-specific culture filtrate from a corresponding strain of *Foc* race 1 (Portal et al., 2018).

Phytotoxins are some of the best studied virulence factors of phytopathogenic fungi and these secondary metabolites can be useful for the prediction of *Foc* virulence on banana plants (Shao et al., 2020) and to improve the existing system for classifying races of *Foc* in future. However, toxins produced by *Foc* race 2 isolates have not been previously identified. This is the first report to explore toxins in a host-specific culture filtrate from a *Foc* race 2 strain.

## Materials and Methods

### Biological Materials

The leaves used in the experiments were sampled from 8 to 9-month-old field-grown banana plants of cultivars “Bluggoe” (*Musa* ABB) and “Grande Naine” (*Musa* AAA), which have a field-behavior of susceptibility and resistance to *Foc* race 2, respectively. Bioassays were done immediately after harvest.

A strain of *Foc* corresponding to VCG 0124 (race 2; kindly provided by Luis Pérez-Vicente, National Center for Plant Health, Havana, Cuba) was used in all studies. The strain was stored as mycelial suspensions in 20% glycerol solution at −80°C until use. Single-spored cultures were recovered on potato dextrose agar (PDA) medium and 1-cm diameter fresh mycelial plugs were transferred to 250-ml Erlenmeyer flasks, each containing 100 ml Czapek-Dox broth with modifications (2 g NaNO_3_, 1 g K_2_HPO_4_, 0.5 g MgSO_4_·7H_2_O, 0.5 g KCl, 0.02 g FeSO_4_·7H_2_O, 20 g glucose and 1 L distilled water). The pH was adjusted to 5.5 with KOH. Cultures were incubated at 25°C in the dark and the cultures from five Erlenmeyer flasks were daily harvested and pooled. The mycelial fresh weight of the five individual cultures was measured every day. Cultures were filtered through four layers of cheesecloth and then centrifuged (8,000 *g* for 10 min) to remove mycelium and conidia. The combined culture filtrates (CFs) were concentrated up to approximately 40% of initial volume by rotatory evaporation under reduced pressure and then were used in the experiments.

### Bioassay

The phytotoxic activity of CFs and extracts (fractions) was assessed by a method described earlier (Companioni et al., 2003) over susceptible (“Bluggoe”) and resistant (Grande Naine) genotypes. Briefly, the third fully expanded leaves were gently collected. The leaves were washed in an abundance of tap water and then washed with sterile distilled water, and blotted on a filter paper. Nine wounds 3-cm apart in the leaf lamina and in between vascular tissue were made by puncturing the leaf with a sterile sharp needle. Wounds were flooded with 5 μl sample or 5 μl uninoculated culture medium (control treatment). CFs or fractions (2 mg each) were solved in 20 μl of methanol and then added to 180 μl of water; the final sample concentration was 10 mg ml^−1^. Leaves were incubated at 26 ± 2°C, 12-h photoperiod, 58 μmol m^−2^ s^−1^, and 70% RH for 48 h. Lesion area was then measured. Each experimental treatment consisted of three plants per cultivar and three leaves per plant. One hundred and forty-four spots were evaluated per treatment. The experimental designs were completely randomized.

### Extraction of Phytotoxins

Toxins were isolated from a host-specific culture filtrate and partially purified based on procedure described by Portal et al. (2018) with minor modifications, as follows. A 29-day-old culture filtrate (10 L) was harvested and concentrated under reduced pression at 45°C using rotary evaporation to approximately 30% of the original volume. An equal volume of ethanol was added and allowed to precipitate overnight at −20°C. The precipitate was collected after centrifugation at 10,000 g for 20 min and the pellet was then resuspended in 5 ml of 10% dimethyl sulfoxide (DMSO) in distilled water (aqueous fraction) before analysis. The supernatant was vacuum-evaporated at 45°C until the volume was reduced to 300 ml. The concentrated CF was successively partitioned between hexane (3x, 2:1, v/v), ethylacetate (3x, 2:1, v/v), and butanol (3x, 1:1, v/v), to yield the corresponding low (154.3 mg), medium (685.6 mg), and high polarity (90.2 mg) fractions, respectively. Extractions were also done from uninoculated Czapek-Dox broth. Aliquots from each fraction were analyzed by thin layer chromatography (TLC). Analytical TLC was done on Merck TLC aluminum-backed silica gel (60 F_254_) plates and the development was carried out using chloroform-methanol-water (14:7:0.5, v/v) as the optimized mobile phase. The plates were dried under room temperature and then dipped in a solution of phosphomolybdic acid (40 g L^−1^) and ceric sulphate (5 g L^−1^) in sulfuric acid (5%), followed by drying with gentle stream of warm air. High performance liquid chromatography (HPLC) grade solvents were used in the various extraction and analytical procedures. Fractions from both uninoculated culture medium and 29-day-old CF were bioassayed as previously described.

### Standards and Reagents

The standards toxins were selected because their occurrence in *Fusarium* species (Jennings, 2007). Mycotoxins used as standards included FA, BEA, enniatin A (ENNA), fumonisin B1 (FB1), moniliformin (MON), deoxynivalenol (DON), and zearalenone (ZEA). Stock solutions of mycotoxins (1 mg ml^−1^) were prepared in methanol and maintained at −20°C. Analytical standards toxins with purities ≥97%, DMSO as well as HPLC-solvents and UV-spectroscopy-grade methanol were purchased from Sigma-Aldrich (St. Louis, MO, United States). Water was successively purified by a Millipore Milli-Q system (Millipore, Bedford, MA, United States).

### HPLC Analysis

The identification of main phytotoxins was done with a LC-MS system. The chromatographic separation of the compounds was carried out on a Restek Ultra C18 column (250 × 4.5 mm, 5 μm) with a Restek guard-column (10 × 4.0 mm). Two solvent mixtures were used as mobile phases. Solvent A was composed of water/methanol (99.2:0.8, v/v and acidified with formic acid 20 μl L^−1^) while 100% methanol was used as solvent B. Samples were dissolved in minimum quantity of methanol. The sample injection volume was set at 20 μl. A solvent gradient was adopted for a total run time of 60 min at a flow rate of 1 ml min^−1^, with all the toxin standards eluting over 20–60 min. The solvent gradient was as follows: equilibration at 5% B for 2 min, from 5 to 35% B in 43 min., from 35 to 100% B in 5 min., 100% B for 10 min., return to initial condition in 2 min.

### Mass Spectrometry

Mass spectrometry analyses were made on a 500 MS Ion trap Mass Spectrometer Varian (Palo Alto, United States) and a Varian Prostar 330 PDA photodiode array detector. An electrospray ionization probe (ESI) in positive ionization and centroid mode was used in the analyses. Conditions were: T° capillary = 350°C, sheath gas flow = 70 meanwhile sweep gas flow = 25, source volt = 5kv, capillary voltage = 70 V, in the range *m/z* 50–1,500; software acquisition and monitoring: X_CALIBUR_ 2.0 SR2.

### Protein Concentration

The concentration of the aqueous extract (pellet) was established as its protein content, which was measured by the method of Bradford (1976) using bovine serum albumin as a standard.

### Statistical Analysis

Statistical analyses of the data were done in the statistical program package SPSS 20.0 (SPSS, Chicago, IL, United States). All values were presented as mean values ± SDs. Statistically significant differences were evaluated by one-way ANOVA followed by Tukey’s test, and *p* < 0.05 was taken to indicate statistical significance.

## Results

### The Phytotoxicity and Specificity Depend on the Age of the Culture Filtrates

**Figure 1:**
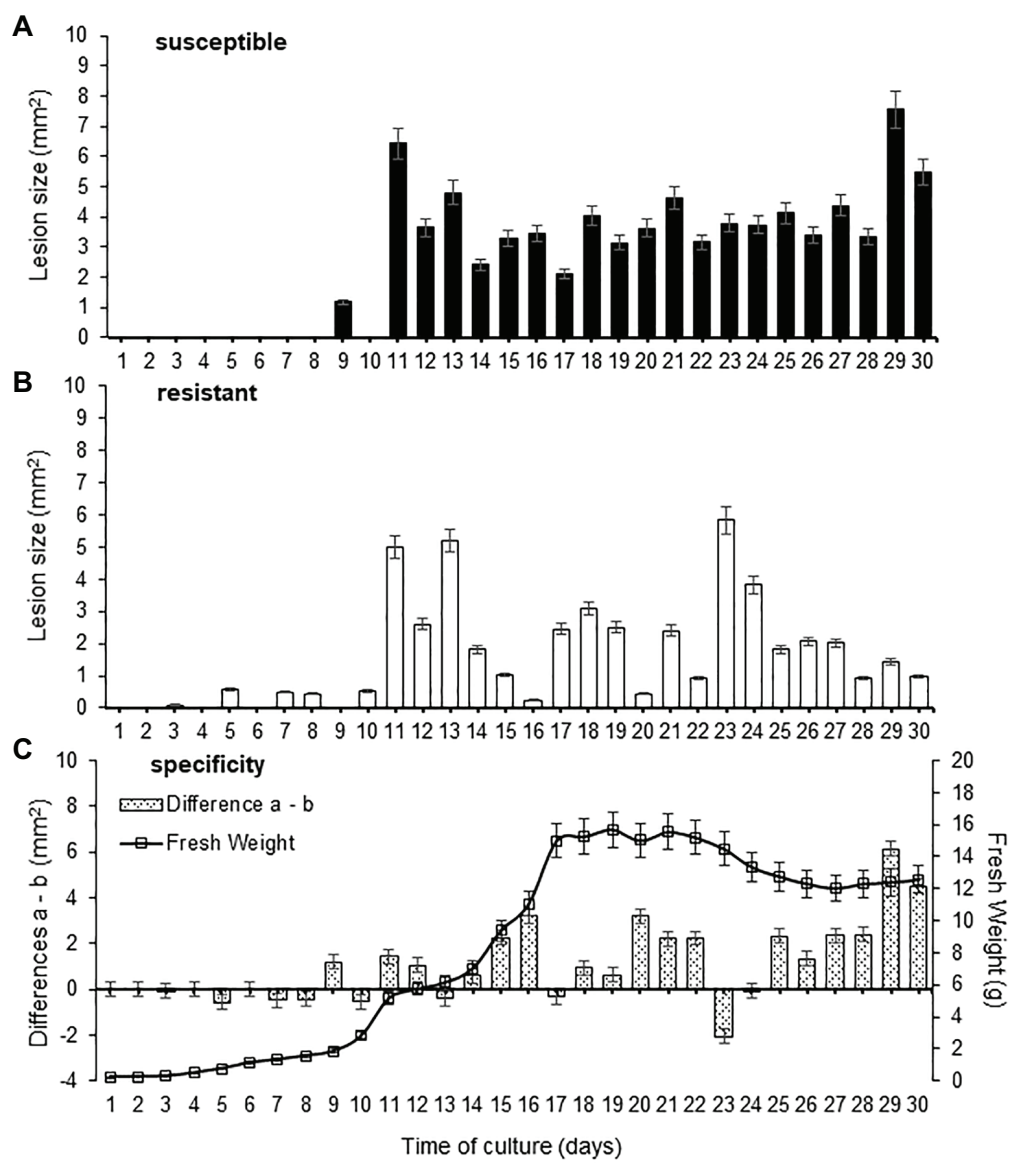
Effect of culture age in fresh weight and phytotoxicity of *Fusarium oxysporum* f. sp. *cubense* (*Foc*) race 2. The leaf lesion size was determined in “Bluggoe” **(A)** and Grande Naine **(B)** leaves after application of the culture filtrates. Also, the specificity of culture filtrates was evaluated according the lesion area between resistant and susceptible clones **(C)**. Bars represent the mean values and SE of the results obtained from three replicates. The experiment was independent replicate three times.

The fresh weight of *Foc* race 2 increased significantly after 9 days of culture reaching a maximum at 17–23 days of culture in modified Czapek-Dox liquid medium (Figure 1C). However, continuous phytotoxicity over leaf tissues for both susceptible and resistant clones were appreciated after 11 days of culture (Figures 1A,B). Despite the continuous production of phytotoxic activity during growth of *Foc* race 2, processes of synthesis and turn-over of microbial metabolites involved on plant tissue response cannot be avoided. The reactions of the resistant and susceptible clones differed over the culture time course (Figure 1C). The highest host-specific effect was achieved with the 29-day-old culture filtrate (time of culture) and, therefore, was selected for further analysis. No lesions were noted with application of Czapek-Dox liquid culture medium alone.

**Figure 2:**
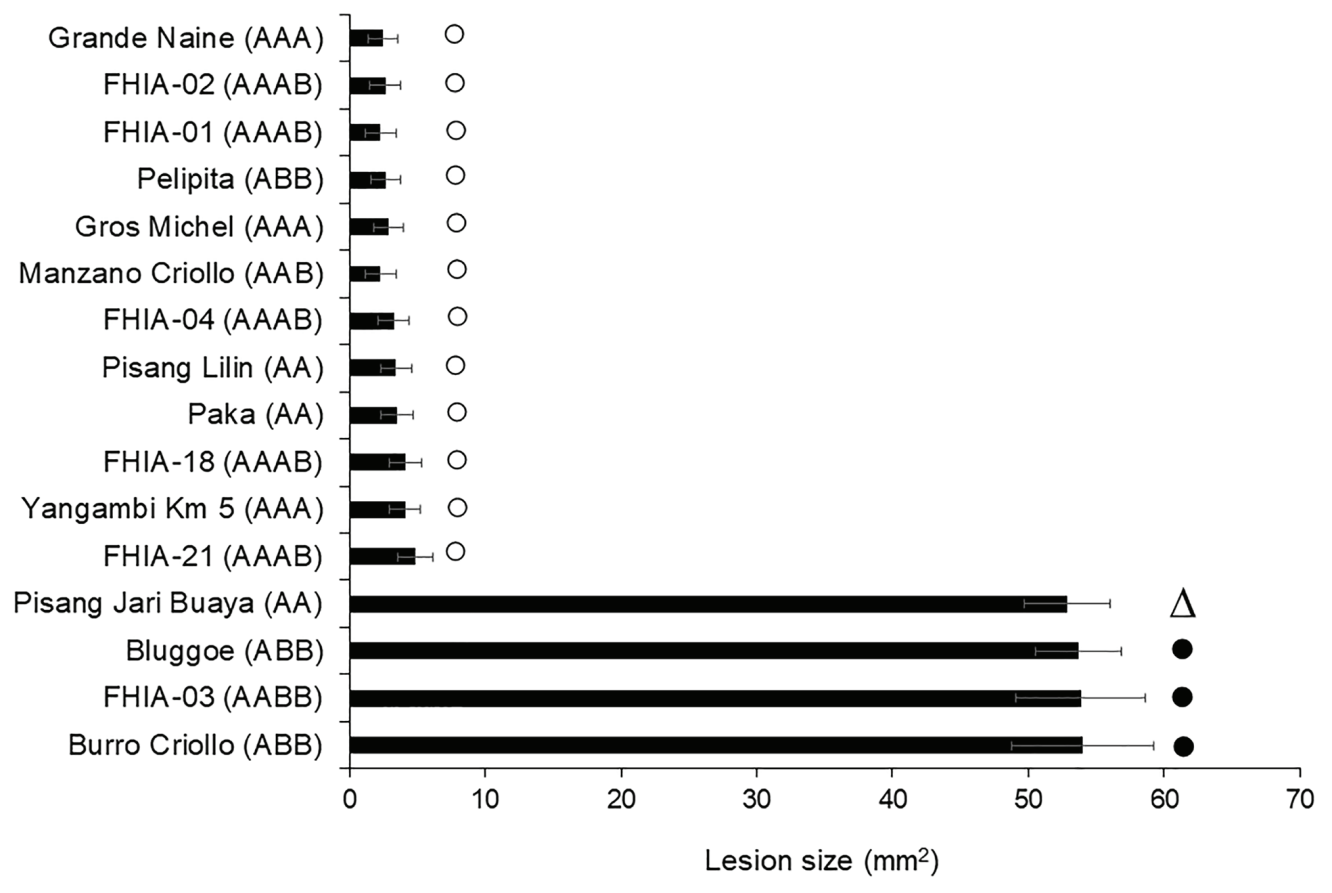
Phytotoxicity of culture filtrate on different plant genotypes with differential disease reaction to Fusarium oxysporum f. sp. cubense race 2. Bars marks show the field defense reaction described as susceptible (•), tolerant (∆) or resistant (○) according to Pérez et al. (2003). Bars represent the mean values and standard error of the results obtained from three replicates. The experiment was independent replicated two times.

To confirm the specificity of the 29-day-old culture filtrates, a similar leaf puncture bioassay described previously was used. Comparison among 16 banana clones with different levels of resistance in field, according to Pérez et al. (2003), is shown in Figure 2. The damage (lesion size) of the culture filtrate on the leaves of the susceptible cultivars ranged from 52.82 to 54.01, while for the resistant ones it was from 2.45 to 4.86. There were no statistical differences within each group. Our results are consistent with previous one informed using *in vivo* assay of resistance level, except for the Pisang jari buaya (AA) clone previously referred as tolerant. Thus, a host-specific effect of 29-day-old culture filtrate was appreciated on the cultivars referred to as susceptible and resistant to the pathogen *in vivo*.

### Phytotoxicity Is Recovered in the Low- and Medium-Polarity Fractions

**Figure 3:**
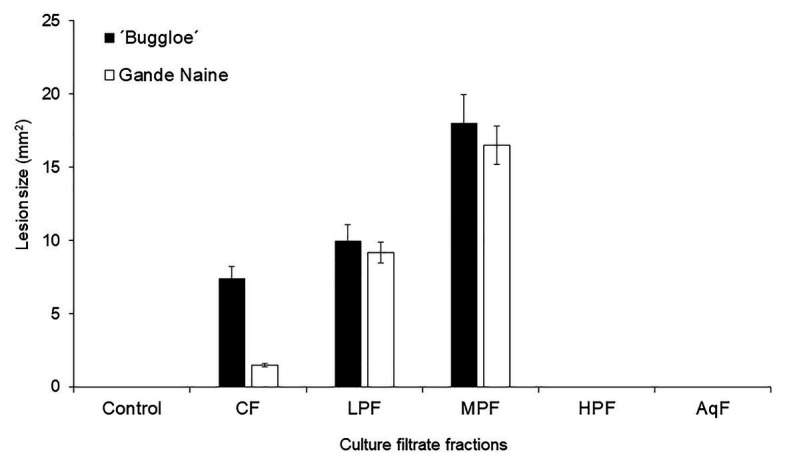
Effect of culture filtrate and its fractions after partitioning with solvents of different polarity from *Foc* race 2. The phytotoxicity (lesion size) was evaluated in resistant (Gande Naine) and susceptible (“Buggloe”) banana cultivars. Control (H_2_O: MetOH); CF (29-day-old culture filtrate); LPF (low-polarity fraction); MPF (medium-polarity fraction); HPF (high-polarity fraction); AqF (aqueous fraction). Bars represent the mean values and SE of the results obtained from three replicates. The experiment was independent replicated three times.

Prior to reveal the identity of phytotoxic metabolites, 29-day-old CF was partitioned between solvents with varying polarities. Total phytotoxic activity was recovered in fraction of low- and medium-polarities. Neither the damage caused by the high-polarity nor by the aqueous fractions was appreciable, while no lesions were detected in solvent/DMSO-treated banana leaves used as controls (Figure 3). However, phytotoxic fractions showed an unspecific effect, i.e., without statistical differences between the damage caused to the leaves of both susceptible and resistant cultivars. This observation suggests the absence of host-specific toxins in a host-specific culture filtrate of *Foc* raza 2.

TLC analysis of the *Foc* race 2 host-specific CF and fractions revealed a similar composition for crude CF, low- and medium-polarity fractions; characterized by the presence of two well-defined spots. The spots occurred as blue and pale yellow, and the retention factors (Rf) were 0.38 and 0.85, respectively. Pale yellow spot eluted at Rf 0.85 was absent in high-polarity fraction, which is clearly associated with phytotoxicity of *Foc* race 2 (Data not shown).

### Identification of Phytotoxins in *Foc* Race 2 Host-Specific CF

**Figure 4:**
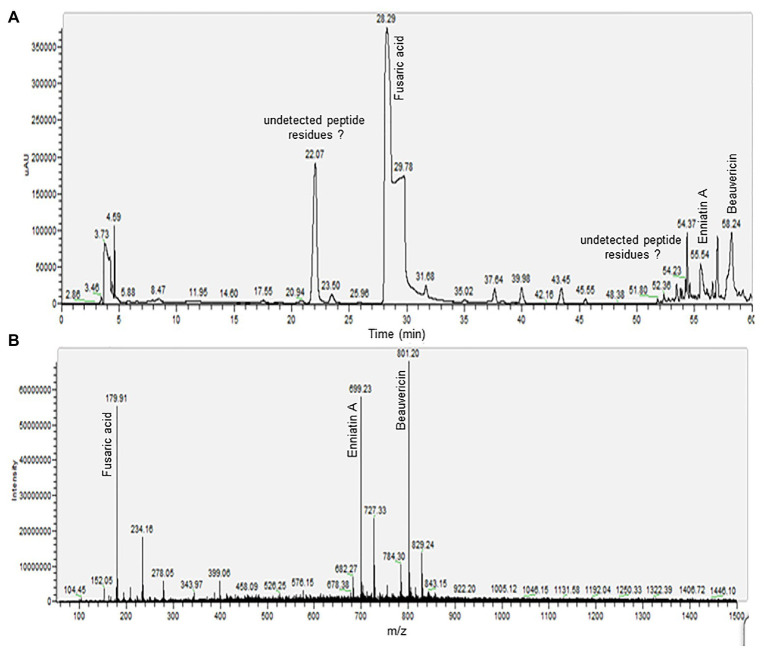
Liquid chromatography electrospray ionization mass spectra (LC-ESI-MS) analysis of phytotoxic fraction from a host-specific culture filtrate of *Foc* race 2. **(A)** High performance liquid chromatography (HPLC) profile of toxins fraction. **(B)** Total ion chromatograms (TICs) generated through ESI-MS analysis in positive ion mode.

The phytotoxic fractions were pooled to obtain toxins fraction, and were later resolved by LC-MS (Figure 4). The LC/MS profile was screened for the presence of mycotoxins by mass comparison with literature data and chemical used as standard.

The electrospray ionization mass spectra (ESI-MS) recording in positive ion mode of the peak eluted at 28.29 min showed the protonated molecular ion signal ([M + H]^+^) for FA at m/z 179.91. On the other hand, the mass spectrum of the peak with retention time of 55.54 min was attributed to ENA, with protonate molecular ion signal ([M + H]^+^) at m/z 682.27, a base peak for a precursor ion [M + NH_4_]^+^ at m/z 699.23 and sodium-adduct [M + 2Na]^+^ ion at m/z 727.33. The compound eluted at 58.24 min was identified as BEA. Pseudomolecular ion ([M + H]^+^) was appreciated at m/z 784.30, a base peak for a precursor ion [M + NH_4_]^+^ at m/z 801.20, and sodium-adduct [M + 2Na]^+^ ion at m/z 829.24. The precursor ion of [M + NH_4_]^+^ for both ENNA and BEA was generated with significant abundance than for [M + H]^+^ and sodium-adduct were also detected for both compounds. Other peaks, such as those eluted at 22.07 and 54.37 min appear to constitute undetected peptide residues in the selected m/z range.

## Discussion

It has been widely accepted that mycotoxins were produced simultaneously with fungal growth, and the rate of production is proportional to its growth rate (Shao et al., 2020). However, our results suggest that the extracellular phytotoxic activity was regulated by the pathogen, occurring during the growth phases and not due to the increased production of mycelial mass. Interestingly, the 23-day old culture filtrate induced greater damage on leaves of the resistant clone than on the susceptible one with a notorious unspecific phytotoxic effect. *Foc* race 2 synthesizes both avirulence determinants [high polarity fraction (HPF)] and nonspecific toxins during its growth. At 29 days, both types of molecules are excreted into the culture medium, while at 23 days, the proteinaceous compounds (HPF) determining the avirulence of the pathogen could be absent in the culture filtrate.

Traditional selection of new banana cultivars under field condition is long-lasting, expensive, with strong influence of the environmental conditions, inoculum diversity, and distribution (García-Bastidas et al., 2019). Also, diverse agronomical factors have a market influence on *Fusarium* wilt of banana under field conditions (Pegg et al., 2019). In contrast, a reproducible bioassay facilitates the evaluation under controlled conditions with specific fungal culture filtrate, leading to reproducible results and accelerating breeding programs (Subramaniam et al., 2006). Therefore, efficient selection agent production constitutes the critical factor in the optimization of phenotyping protocols.

Morpurgo et al. (1994) failed to establish a clear correlation between *in vivo* and *in vitro* plant response when 21-day-old culture filtrates from strains of both *Foc* race 1 and *Foc* race 4 were used as selection agent, suggesting that the use of crude filtrate in a screening program for selecting resistant banana genotypes is not necessarily accurate (Li et al., 2013). However, previous to the isolation of phytotoxins involved in *Foc* race 2 pathogenicity, we obtained a host-specific culture filtrate (29-day-old) from a pathogenic isolate of *Foc* race 2 that allows differentiating resistant cultivars from susceptible ones. In previous report, banana clones resistant to *Foc* race 1 were differentiated from susceptible one using a 15-day-old culture filtrate as selection agent (Portal et al., 2018), evidencing that culture age is important to achieve host-specificity when use culture filtrate as selection agent.

*Fusarium* species colonizes the host plant producing toxins in the necrotrophic stage to take advantage whereas hijack host secondary metabolic pathways (Perincherry et al., 2019). Therefore, identification of host tissue response-associated fungal metabolites from a host-specific culture filtrate contributes to understand the molecular events of isolated molecules in pathogenicity of *Foc*. In that regard, we used the 29-day-old culture filtrate to ensure that the identified compounds play a role in the response of banana plants to *Fusarium* wilt.

However, unspecific effect of partially purified metabolites recovered in low- and medium-polarity fractions suggested other compound(s) rather than phytotoxins as responsible of specificity. Pathogens secrete proteins during colonization to establish a successful pathogenesis. Some secreted proteins are called “effectors,” which promote host colonization, often by modulation of plant immunity. Different effectors can suppress the innate immunity. Meanwhile, others are detected by the plant innate immune system and then activate the defense, changing from virulence to avirulence patterns (Ma et al., 2015). The HPF protected the leaves of the resistant banana cultivar from damage caused by the phytotoxic metabolites contained in a host-specific culture filtrate, suggesting the presence of avirulence determinants. Fusaric acid (5-n-butyl-pyridine-2-carboxylic acid, C_10_H_13_NO_2_) is a non-specific phytotoxin that is produced by many fungal pathogens to cause diseases in plants (Niehaus et al., 2014; Brown et al., 2015; Selim and El-Gammal, 2015). The crude toxin extract from culture filtrates of *Foc* race 4 and pure FA had similar effects as the pathogen infection in Cavendish banana plants. Additionally, the colonization of cavendish plants by *Foc* race 4 was significant higher compared with *Foc* race 1, as was the content of FA in those infected plant tissues (Dong et al., 2019). Fusaric acid played a crucial role in accelerating the senescence of *Foc* race 4-infected plants (Dong et al., 2014) and it has been shown to be essential for *Foc* virulence on banana plantlets (Ding et al., 2018; Liu et al., 2019).

Similar to others secondary metabolite biosynthetic genes which were generally located in gene clusters, the deletion of FoFUB4 gene led to a total loss of FA production and attenuated virulence of a *Foc* race 4 strain compared with WT (Ding et al., 2018). Despite on prominent role played by FA in *Foc* virulence, disease severity caused by mutant ΔFoFUB4 suggest that other virulence factors would be involved in *Fusarium* wilt of banana. Additionally, Shao et al. (2020) argued that the pathogenicity of *Foc* may not be conferred to a single mycotoxin, instead a synergy of several mycotoxins.

Beauvericins and enniatins share a common cyclohexadepsipeptide structure as a basic skeleton. They are ionophoric compounds capable of forming complexes with monovalent and divalent cations through interactions with carbonyl groups oriented within the molecule (Belén Serrano et al., 2015). These biomolecules are produced by many *Fusarium* species (Urbaniak et al., 2019) and has been showed to play an important role in the pathogenicity of *Fusarium avenaceum* in wheat (Logrieco et al., 2002). Also, a putative role of this toxin in phytotoxicity of tomato protoplasts was determined (Paciolla et al., 2004). Only two mycotoxin, FA and BEA, were detected in the 20 *Foc* isolates evaluated, including seven *Foc* race 1 and 33 *Foc* race 4 representatives. Fusaric acid and BEA had a phytotoxic effect in banana protoplast although BEA proved to be significant more toxic than FA (Li et al., 2013).

Enniatins have residues of hydroxyisovaleric acid, isoleucine, valine, and leucine (Bashyal et al., 2019). These toxins produce a complex with metal cations in biological membranes disrupting cation gradients through the cell, affecting the cellular homeostasis. However, ENNs production in *F. avenaceum* alone has not influence in the disease severity of *Fusarium* head blight (FHB) in wheat and peas under greenhouse conditions. Eranthodi et al. (2020) shown that the enniatins offset the action of other metabolites or enzymes related with the pathogenicity.

Up to now, our work shows for the first time the identification of phytotoxic metabolites in a *Foc* race 2 culture filtrates. Fusaric acid, ENNA, and BEA were found to be present in a *Foc* race 2 host-specific culture filtrates. We speculate that while some, if not all, identified phytotoxins are important virulence factors, differences in pathogenicity on specific host cultivars could be due to protein fraction of the host-specific CF, but further investigation is needed.

## Data Availability Statement

The original contributions presented in the study are included in the article/supplementary material, further inquiries can be directed to the corresponding authors.

## Author Contributions

NP and RS conceived the study. NP, CR, JS, and BC performed the experiments and analyzed the data. AS, LH, and RP supervised and provided the suggestion of the research work. RS and OB-H prepared the manuscript. All authors contributed to the article and approved the submitted version.

### Conflict of Interest

RP and OB-H were employed by the company Yota Bio-Engineering Co., Ltd.

The remaining authors declare that the research was conducted in the absence of any commercial or financial relationships that could be construed as a potential conflict of interest.
